# Building the Ferretome

**DOI:** 10.3389/fninf.2016.00016

**Published:** 2016-05-10

**Authors:** Dmitrii I. Sukhinin, Andreas K. Engel, Paul Manger, Claus C. Hilgetag

**Affiliations:** ^1^Department of Computational Neuroscience, University Medical Center Hamburg-EppendorfHamburg, Germany; ^2^Department of Neurophysiology and Pathophysiology, University Medical Center Hamburg-EppendorfHamburg, Germany; ^3^School of Anatomical Science, University of the WitwatersrandJohannesburg, South Africa; ^4^Department of Health Sciences, Boston University, BostonMA, USA

**Keywords:** brain connectivity, connectomics, tract-tracing, ferrets, databases as topic, models, theoretical

## Abstract

Databases of structural connections of the mammalian brain, such as CoCoMac (cocomac.g-node.org) or BAMS (https://bams1.org), are valuable resources for the analysis of brain connectivity and the modeling of brain dynamics in species such as the non-human primate or the rodent, and have also contributed to the computational modeling of the human brain. Another animal model that is widely used in electrophysiological or developmental studies is the ferret; however, no systematic compilation of brain connectivity is currently available for this species. Thus, we have started developing a database of anatomical connections and architectonic features of the ferret brain, the Ferret(connect)ome, www.Ferretome.org. The Ferretome database has adapted essential features of the CoCoMac methodology and legacy, such as the CoCoMac data model. This data model was simplified and extended in order to accommodate new data modalities that were not represented previously, such as the cytoarchitecture of brain areas. The Ferretome uses a semantic parcellation of brain regions as well as a logical brain map transformation algorithm (objective relational transformation, ORT). The ORT algorithm was also adopted for the transformation of architecture data. The database is being developed in MySQL and has been populated with literature reports on tract-tracing observations in the ferret brain using a custom-designed web interface that allows efficient and validated simultaneous input and proofreading by multiple curators. The database is equipped with a non-specialist web interface. This interface can be extended to produce connectivity matrices in several formats, including a graphical representation superimposed on established ferret brain maps. An important feature of the Ferretome database is the possibility to trace back entries in connectivity matrices to the original studies archived in the system. Currently, the Ferretome contains 50 reports on connections comprising 20 injection reports with more than 150 labeled source and target areas, the majority reflecting connectivity of subcortical nuclei and 15 descriptions of regional brain architecture. We hope that the Ferretome database will become a useful resource for neuroinformatics and neural modeling, and will support studies of the ferret brain as well as facilitate advances in comparative studies of mesoscopic brain connectivity.

## Introduction

### Connectomics

A central perspective for analyzing brain data is the representation of neural relations as complex networks. This representation can be used for almost all structural-functional dimensions of the brain, from the molecular to the systems scale, and structural to cognitive characterizations. The network-theoretical approach is a powerful tool in the hands of neuroscientists, because it provides a formalized framework for the analysis of complex interactions (Klimm et al., 2014). In particular, different types of brain connectivity can be distinguished, such as functional connectivity (reflecting statistical dependencies among neurophysiological events) as well as effective (causal) connectivity (Friston, 1994). The most fundamental type of connectivity is structural or anatomical connectivity, which provides a structural network basis of brain dynamics and function.

Several current projects address the challenge of collating the complete structural network of the brain, the so-called connectome (Sporns et al., 2005), from the cellular to the mesoscopic and macroscopic scale (Leergaard et al., 2012). The neuronal micro-connectome, which is based on invasive methods of imaging and the reconstruction of neuronal elements (including synapses) from brain sections (see Van Essen et al., 2013 for an extensive review), may form the ultimate structural basis of the brain. However, connectomics at the cellular level faces a host of conceptual and technical challenges and cellular connectomes have so far only been completed for the small nervous systems of the nematode *Caenorhabditis elegans*, possessing just 302 neurons (White et al., 1986; Varshney et al., 2011), as well as partly for neural populations in the zebrafish (Friedrich, 2013) and *Drosophila* (Chiang et al., 2011; Shih et al., 2015). One of the main problems of constructing connectomes at the microscopic level is the computationally demanding reconstruction of synaptic connections from the raw data that places limitation on the volume of brain tissue that can be studied (Helmstaedter et al., 2008). Recently, considerable progress to overcome these limitations has been made in terms of methodology (reviewed in Kleinfeld et al., 2011), resulting in advances that may eventually lead to the creation of a whole connectome of the mouse brain (Mikula et al., 2012). Moreover, by applying new methods from genomics, it might be possible to create micro-connectomes for a wide range of species (Zador et al., 2012).

Examples for connectomes at the macroscopic level include the recently published data on brain-wide mouse connectivity (Oh et al., 2014; Zingg et al., 2014), partly based on optogenetic methods for labeling and tracing axonal connections of large-scale regions of interests (that is, cortical areas and subcortical nuclei). Further anatomical tracing techniques can be used to obtain structural connectivity at the mesoscopic level. The conventional method of histochemical tract-tracing has produced significant insights into the organization of brain connectivity and has resulted in an extensive body of connection data, for example, a detailed description and analysis of macaque monkey visual cortical connectivity (Felleman and Van Essen, 1991) and connectivity of the entire mesoscopic cat cortical (Scannell et al., 1995) and thalamocortical system (Scannell et al., 1999) as well as extensive connectivity of the rat at the systems level (Bota et al., 2015). These connectivity data were compiled from traditional neuroanatomical studies performed during the last decades. As a further attempt to systematize this approach of generating structural connectivity, and in order to deal with methodological problems such as different parcellation approaches and methods of labeling, connectivity databases such as the CoCoMac database were created (Stephan et al., 2001; Bakker et al., 2012; Stephan, 2013). Over a period of more than 10 years, hundreds of tract-tracing reports for the macaque monkey brain were collated in CoCoMac (Bakker et al., 2012).

A fundamental problem of conventional anatomical tract-tracing studies is that, due to their invasiveness, they cannot be performed in humans. This limitation raises questions about the applicability of data gathered in the animal models to humans. The problem can be ameliorated by comparative studies of different animal models (Bohland et al., 2009; Goulas et al., 2014; Zingg et al., 2014; Bota et al., 2015), and through newly developed non-invasive techniques for imaging connectivity-related parameters. For example, diffusion imaging methods such as diffusion tensor imaging (DTI) or diffusion spectrum imaging (DSI) can be used to produce entire connectomes of a human brain in relatively short time (Van Essen et al., 2012). Diffusion imaging measures the anisotropy of water diffusion along axonal paths, which can then be used to infer the course of fiber tracts. The approach is systematically exploited by large-scale projects such as the Human Connectome Project (Toga et al., 2012), which aims to provide a comprehensive description of all long-range pathways of the human brain. However, diffusion-based approaches may be prone to several measuring and reconstruction artifacts (Farquharson et al., 2013).

The rise of new imaging methods such as DTI raises the question of whether connectivity databases based on laborious and invasive anatomical tract-tracing studies are still required. The answer should be affirmative, as such conventional data provide a well-established ‘gold standard’ of structural brain connectivity. With this approach, one can directly observe the labeled origins and terminations of projection neurons in different brain regions, gather information on the axonal density and direction of projections as well as finer details, such as the laminar origins and terminations of projections. All of these aspects, which may be of substantial functional importance (e.g., Vanduffel et al., 1997), are currently not accessible by diffusion-based tractography.

It should, however, be noted that conventional anatomical tract-tracing studies are not without potential technical and methodological problems either, considering, for example, mislabeling due to the spillage of tracer injections into neighboring regions or the white matter (for further discussion of these issues see Kötter, 2001). Moreover, there are also challenges associated with the many alternative ways of parcellating the brain into different areas, by not completely objectified criteria. For example, brains may be parcellated by using various multi-modal macroscopic or cytoarchitectonic criteria (Dombrowski et al., 2001; Amunts et al., 2014), as well as personal preferences. One way to address these problems is through knowledge management methodology. One current project in this field is Neurolex (Neurolex.org; Larson and Martone, 2013) which allows to organize and query neurobiological knowledge by inter-referencing and linking it to detailed empirical data. A further example is UBERON^1^, which provides cross-species hierarchical parcellations of regions of interest of the nervous system. However, due to the cross-species generality of the approach, the annotation is rather coarse, as contrasted with detailed existing parcellations in an individual species such as the ferret, which, for structures such as the cerebral cortex, already possess several dozen parcels. Therefore, the practical value of this systematic approach for the current project is limited. Generally, despite the obvious advantages of a systematic organization of neurobiological knowledge for the scientific community, advances in knowledge management methodology are still mostly ignored by the authors of tract-tracing reports (see Bakker et al., 2012 for review).

In addition, many reports in the literature do not provide quantitative data on the number of labeled neurons or the numerical density of axonal terminations, but only categorical information on the presence or absence of pathways, or comparative qualitative measures, such as ‘low’/ ‘average’/ ‘high’ density of connections (Lanciego and Wouterlood, 2011). This type of coding may encompass a great range of quantitative values. For example, the density of anatomical pathways (that is, the number of axons in them) can extend over five orders of magnitude (Markov et al., 2011) and may be poorly captured by a limited number of ordinal categories.

### The Model System of the Ferret Brain

Due to limitations of directly investigating the structural connectivity of the human brain, research has turned to animals models, where extensive developmental, behavioral, or electrophysiological data can be obtained. Here, the ferret brain has some distinctive advantages. For example, one benefit in developmental studies is the convoluted, gyrencephalic surface of the ferret brain and that the process of gyrification can be observed in detail (Sawada and Watanabe, 2012). Immaturity of the ferret at birth helps to investigate developmental processes that occur prenatally in other species, such as the cat, and, for example, allows conducting systematic experiments with altered connectivity in order to observe the adaptation of cortical areas to new sensory stimuli (Noctor et al., 2001). Moreover, the relative developmental immaturity of the neonatal ferret facilitates studies on how early lesions in one part of the brain may affect connectivity in other regions (Restrepo et al., 2002), and how lesions have an impact on the development of topographical maps and connectivity between the cerebral hemispheres (Restrepo et al., 2003). A further advantage of the ferret is that its brain shows substantial homologies with other species, such as the cat (Manger et al., 2010) as well as potentially with other carnivores such as the dog (Onishi et al., 2007). Taking these factors into account, extensive work has been performed in this animal model using electrophysiology to relate patterns of electrical activity to behavior (e.g., Fritz et al., 2003; Bizley et al., 2013). These studies have shown that ferrets possess intricate sensory cortical systems (Phillips et al., 1988; Nelken and Versnel, 2000; Innocenti et al., 2002; Bizley et al., 2005, 2007; Manger et al., 2005), making them an appropriate model for the study of sensory processing pathways, response properties and topographies of sensory neurons and multisensory interactions. In fact, there exists no comparable model at the moment that combines elaborate and easily trainable behavior with the opportunity for extensive anatomical and physiological as well as developmental studies. In particular, similar studies in primates, which proceed only in very few labs, are much more restricted in the scope of investigations and the number of animals studied.

In addition to the advantages of the ferret brain model for anatomico-physiological research, one should also point out its usefulness for comparative studies. Currently, systematically compiled macro-connectivity data are only available for a restricted range of species (macaque monkey, cat, rat, and mouse) limiting the ability of cross-species analyses. Extending the number of available connectomes of different species for systematic statistical and graph theoretical analyses can shed light on the general organization of connectivity patterns in mammalian brain networks (Striedter, 2005). One successful example of such inter-species comparisons is the identification of a densely connected ‘rich club’ of core brain regions in different species (van den Heuvel and Sporns, 2011; Harriger et al., 2012; Towlson et al., 2013) and its role in brain diseases (van den Heuvel et al., 2013).

Hence, a detailed macroconnectome of the ferret brain will facilitate comparative anatomical studies and support cross-domain exchange in anatomy, electrophysiology and connectomics. Another specific motivation of the ferretome project is to provide data for the connectivity-based modeling of ferret brain dynamics. This modeling project is part of a research collaboration with experimentalists recording brain activity at multiple sites of the ferret brain using ECoG and multi-electrode approaches (Stitt et al., 2015a,b). As a necessary precondition for the modeling, the structural connectivity of the ferret brain as well as further features of its brain architecture need to be known. However, at the moment, no systematic compilation of connectivity is available for this species. Creating a repository of the macroconnectivity of the ferret brain is a complex task. The collation of the data from published tract-tracing report faces similar problems as previously addressed by the CoCoMac database (Bakker et al., 2012) or projects such as BAMS (Bota et al., 2005, 2015) and neuroVIISAS (Schmitt and Eipert, 2012). Thus, in the following section we provide a short review of existing database projects that aim at storing connectivity data, in order to define the parameters of a suitable architecture for the ferret brain connectivity database.

## Comparable Work

In the area of connectivity databasing, two main types of approaches for representing brain topography can be distinguished: coordinate-based vs. semantic or logical parcellation schemes. The first type is represented by the XANAT system (Press et al., 2001), while the second approach is used by the remainder of projects reviewed below.

XANAT (Press et al., 2001) was one of the first systems for storing, comparing and analyzing the results of neuroanatomical connection studies. Data can be entered into the system by placing injection and label sites into canonical representations of the neuroanatomical structures of interest, along with verbal descriptions. After the entry procedure, a graphical search can be performed on the data by selecting a specific brain site or textual search with use of keywords or references to original studies. An important feature of the system is that data may be studied and compared relative to well–known neuroanatomical substrates or stereotaxic coordinates regardless of variable areal boundaries (Press et al., 2001). XANAT can be downloaded and run in the Unix X window environment (as reflected in the name of the software).

The brain architecture management system (BAMS) (Bota et al., 2005, 2015), is a representative example of the attempt to store comprehensive structural descriptions of the brain. Information about four main entities and their attributes can be kept in the system: connections, relations, cell types and molecules. The connections entity represents records of data and metadata of macroscopic neuroanatomical projections between brain regions. The relations entity describes qualitative spatial relations between brain regions. Cell type attributes provide descriptions of neurons, neuronal population and their classifications. The molecules category represents data on molecules (e.g., neurotransmitters) specific to neurons and brain regions.

BAMS is accessible online via a web interface^2^ The server part is written in PHP^3^ and the database itself is handled by MySQL^4^ In BAMS, data can be stored and found for different species; however, the majority of it reflects structural descriptions of the rat. Some data can be exported for further analysis in structured formats (for example, as an adjacency matrix).

A further system, the NeuroVIISAS platform (NeuroVisualization, Image mapping, Information System for Analysis and Simulation; Schmitt and Eipert, 2012) is an example of a neuroinformatics approach that aims to link the storage of connectivity information with visualization and analysis. NeuroVIISAS is an open framework that allows users to perform integrative data analysis, visualization of the data, and even population simulations with the help of a link to the NEST software for neuronal simulations (Gewaltig and Diesmann, 2007). During the data analysis step, it is possible to use a variety of network manipulations, such as network randomization and comparisons to benchmark networks (e.g., scale-free networks). Connectivity matrices can be visualized together with summary indices for characterizing brain connectivity, such as the clustering coefficient (Holland and Leinhardt, 1971) or the joint degree distribution (Albert and Barabási, 2002). Visualization, in particular of rat connectivity, can be provided in the framework of the Paxinos and Watson (2006) atlas. Population simulations based on the connectivity data can be performed using PyNEST (Davison et al., 2008) and NEST (Gewaltig and Diesmann, 2007). In this way, neurobiologically defined connectivity is integrated with computational neuroscience simulations. After script generation and simulation, the produced results can be imported back into NeuroVIISAS and visualized in various formats, including 3D visualization. NeuroVIISAS is a free software implemented in Java with versions for Windows and Linux, which can be operated locally. The main advantage of this approach is that a researcher’s own data (connectivity or mapping information) can be quickly added to the framework and analyzed, visualized, and simulated in the local environment.

Finally, CoCoMac (Collation of Connectivity data on the Macaque brain) is a connectivity database and neuroinformatics platform that has been developed for more than a decade (Stephan et al., 2001; Bakker et al., 2012; Stephan, 2013). CoCoMac aims to store two main modalities of data: connectivity tract-tracing studies as well as mapping studies of (mainly) rhesus macaque. CoCoMaC addresses central challenges of collations of connectivity from the anatomical literature, such as the absence of spatial coordinates in many primate anatomical studies and of a universally accepted brain map for the Macaque monkey. These aspects result in inconsistencies between alternative brain parcellation schemes, as well as ambiguities and contradictions of results from different tract-tracing studies. The CoCoMac creators postulated five main principles for their project: Objectivity, Reproducibility, Transparency, Flexibility, and Simplicity. These principles reflect the way in which the system links to original data, as well as the schema by which data are inserted and processed in the database. In particular, a specific algorithmic framework was developed, termed objective relational transformation (ORT; Stephan and Kötter, 1999; Stephan et al., 2000b). This framework allows the transformation of all available connectivity data in one brain map into another map, according to relations between areas and brain maps established in the anatomical literature, using an encoding of logico-spatial relations between the regions (e.g., an area A is smaller than, bigger than, equal to, or overlaps with, another area B).

Originally, CoCoMac was created in MS Access, but subsequently the database was converted to MySQL and made accessible through a web interface, with the server side programmed in PHP. With the update to a new version^5^, CoCoMac received several new features including a search/browse wizard and direct access to the database content through specifically developed viewers (Bakker et al., 2012).

In summary, in this section we reviewed existing neuroinformatical approaches for representing experimentally established brain connectivity as a network model at different scales. Despite the rise of new experimental methods, such as DSI/DTI, at the macroscopic level, anatomical tract-tracing studies are still the most reliable source of connectivity data. Availability of macroscopic connectivity data for a variety of species will facilitate comparative studies and deepen our understanding of the particular organization of the human brain. One popular animal model is the ferret due to its valuable features, such as elaborate behavior and immaturity at birth. Creating a complete brain connectivity scheme of an animal even as small as a ferret is a complex task that requires the help of modern methods in computer science such as online databasing. In the next section we turn to the issue of building such a database, populating it with data, supporting it and extracting summary results.

## Methods

### Basic Design

From a conceptual point of view, the main structure of the Ferretome database was derived from the CoCoMac project (Stephan et al., 2001). The CoCoMac data model allows the storage of three data modalities: mapping information, labeling data, and meta data about brain map relations as well as special data codes.

**FIGURE 1:**
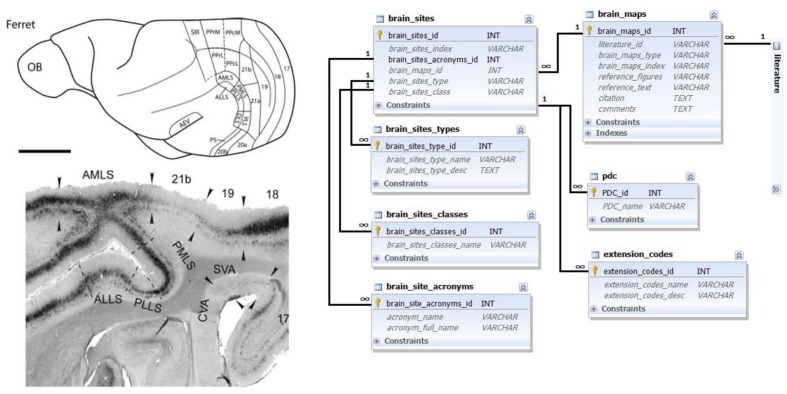
**Brain map data and its representation in the database.** Left top: schematic ferret brain map delineation; left bottom: detailed brain map delineation on a microphotograph of a stained brain section; both panels reproduced with permission from Homman-Ludiye et al. (2010). A brain map represents a set of delineated areas with characteristic names described in the figures or text of literature references. Right side: Ferretome.org database schema related to the brain maps data modality. The main entity is a brain map (linked to a literature table). One brain map can encompass many brain sites with different acronyms and types. Brain areas can, for example, be classified into cortical and subcortical regions, and supplemented with special data codes (PDC and extension codes, described in the main text).

Mapping information is based on published verbal or graphic descriptions of brain parcellations, structuring the brain into multiple areas and nuclei, typically according to the characteristic architectonic or physiological properties of these parcels (see **Figure 1** for illustration.)

**FIGURE 2:**
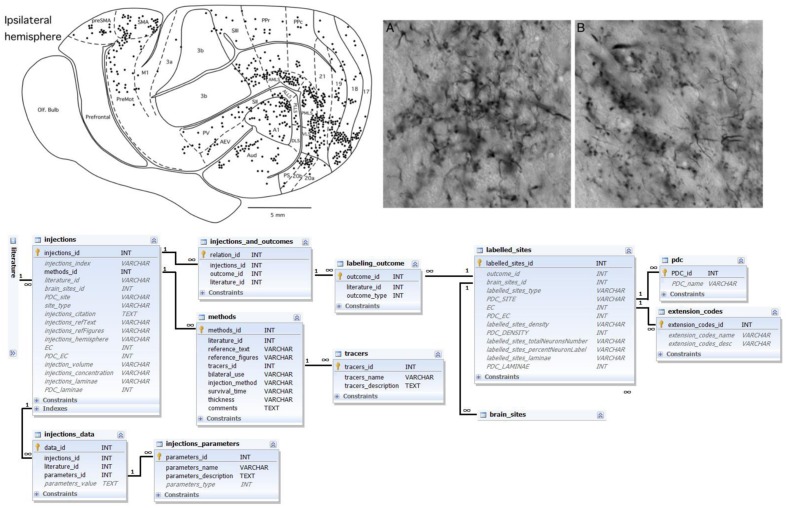
**Labeling data and its representation in the database.** Top left: schematic representation of the outcome of a connection labeling experiment in the ferret brain; top right: corresponding microphotographs of stained sections and labeled neurons; both panels reproduced with permission from Manger et al. (2010). Bottom: Ferretome.org database schema related to the data modality of connection labeling experiments. The central entity of this schema is an injection (linked to a literature table). One connection-tracing report may comprise several injections. Many injections have several outcomes. Every outcome comprises many labeled sites that should be linked to brain sites (cf. **Figure 1**). All injections are supplied with data about methods, tracers as well as further parameters.

Connection labeling information is based on verbal or graphic descriptions of results of labeling experiments. In the tract-tracing literature, the results of connection labeling experiments may be published together with their own mapping scheme or use previously published maps. In both cases, a tract-tracing experiment describes locations of tracer injections (injection site – a brain area in a specific brain map or part of a brain region, e.g., “caudal parietal cortex”) and locations where tracer was found (labeled sites). The density of the label is usually coded in a qualitative parameter – from ‘weak’ to ‘strong.’ Further information about the tract-tracing methodology may be given (for example, the number of studied animals, type of tracer and its amount, survival time of animals and thickness of brain sections that were evaluated). See **Figure 2** for details.

Meta information can be divided into two main types. The first type concerns brain map relations. This type of data is published in its own right or provided as part of tract-tracing studies and usually given as a verbal description of how brain areas in one parcelated map are related to brain areas in another map. Across the tract-tracing literature, five main relations of brain areas can be found. Brain areas can be identical, area A may be a subarea of area B, two areas can overlap with each other, area B may be a subarea of A, or the areas may be unrelated.

As a second type of meta information, the creators of CoCoMac introduced special descriptions in order to cope with issues of data ambiguity and lack of data. The first of these descriptions is the “Extension code.” This code describes the extent of information available for a brain area or a labeled site. This code has several states: information may be available for an entire brain site, part of a brain site or for no part of a brain site. This code is used subsequently by the algorithmic engine of CoCoMac.

A further kind of characterization is given by the so-called precision data codes (PDC). PDCs were used in CoCoMac in order to cope with situations where the information contained in the text of a paper apparently contradicts information in figures or tables. Here, the PDC is coded by letters from “A” to “Q,” where “A” stands for the most reliable and consistent description. For example, the PDC code “A” for specifying a brain area signifies that “The area is named explicitly in the text/tables and identified with certainly. Additional figures explicitly support the text by showing present (or missing) label in areas defined by names and/or borders”, whereas “Q” indicates: “The information about the (un)labeled area is not from an original research report, but from a review article” (more details can be found in Stephan et al., 2001). CoCoMac provides several types of PDC’s for different types of data, for example, PDC_BrainArea, PDC_lamina, with their own specific descriptions.

**FIGURE 3:**
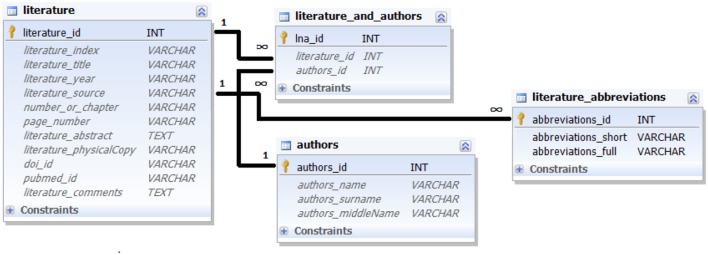
**Ferretome.org database schema for literature references.** The schema captures the essential bibliographic information.

All three data modalities can only be entered into the database together with links to a concrete data source. For this purpose, CoCoMac and Ferretome.org provide special tables to store information on literature references and their authors (**Figure 3**).

Another distinctive feature of CoCoMac is the incorporation of the approach of ORT (Stephan et al., 2000b). This powerful algorithm allows the automatic conversion of all available data (including PDCs) from one given brain map into another. ORT uses a custom-developed relational algebra that handles the five main relations between brain areas, as mentioned above: identical, subarea, larger, overlap and disjoint (for details see Stephan et al., 2000b). Specifically, if there exists a report that specifies a relation among brain maps, then it is possible to transform connectivity data from one report to another and hence to build a consistent description of brain connectivity. For example, if two areas from two different brain maps are specified by a report as “identical,” then all data associated with these areas can be easily transferred from one map to another. In addition to transforming data for known relations among brain maps, ORT is capable of discovering previously undefined relations between brain areas of different maps (i.e., which are not yet specified in the anatomical literature). For example, if it is known that “A” is identical to “B” and “B” identical to “C,” it can be inferred that “A” is identical to “C.” The algorithm can also identify inconsistent relations (such as that “A” is a subarea of “B” while also “B” is a subarea of “A”).

### Extending the Basic Design

In creating the Ferretome database, the template data model and algorithmic services of CoCoMac were adjusted to species-specific properties of the ferret brain as well as additional requirements established during the conceptual planning.

The main novelty, in terms of the database structure, was the introduction of extensible and flexible tables that store data about ferret brain architecture and the means to process this data as part of the standard data model. After an extensive review of presently available reports on ferret brain architecture we found that this new data modality has several distinct features. For example, architecture parameters can be applied to a whole area or part of an area. Such parameters can be quantitative as well as qualitative. For instance, quantitative data may exist on primary and secondary cell diameters, the number of layers and sublayers and their thickness. Alternatively, one may find qualitative descriptions of CO reactivity, myelination (e.g., in terms of “weak,” “average,” “strong”), laminar differentiation and types of neurons and their sizes (e.g., “big pyramidal neurons,” “small granular neurons”).

**FIGURE 4:**
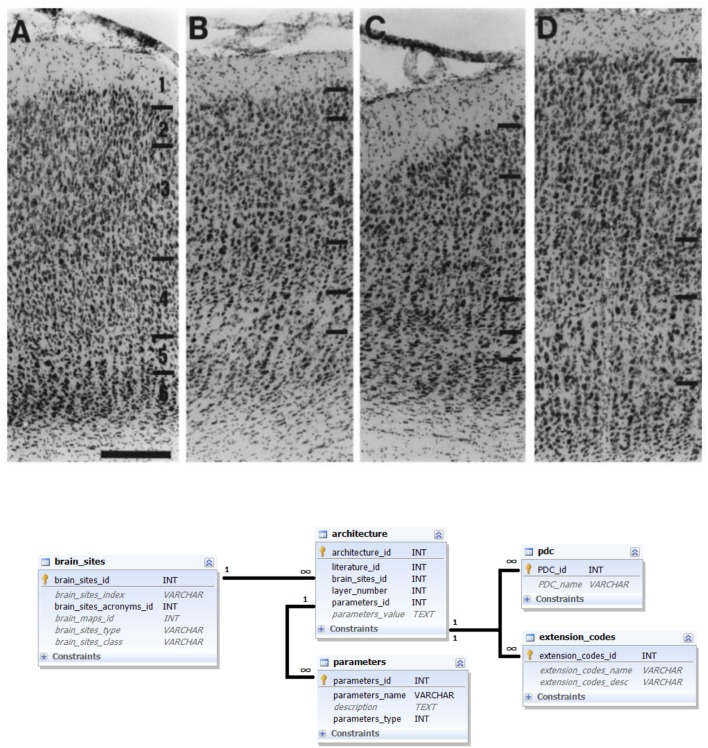
**Architecture data and its representation in the database.** Upper panel: Example of a literature report on brain architecture data. Photomicrograph of stained sections of several brain areas (from left to right: areas 17, 18, 19, and 21) with indicated laminar subdivisions and 200 μm scale bar (Innocenti et al., 2002); reproduced with permission. A corresponding textual description reads: “Area 17. In Nissl stains […], layers are easily delineated. Layer 2 consists of small, round cell bodies, more densely packed than in layer 3, which is characterized by both small and medium- sized pyramidal cells. Layer 4 is thick and can be subdivided into 4a and 4b, characterized by large granule cells, and 4c, which is thinner and consists of smaller granule cells. Layer 5 has the lowest cell density and contains large pyramidal neurons. In layer 6 the cell density increases again and the neurons are organized in distinct, radially oriented palisades, 2–3 cell bodies thick” (Innocenti et al., 2002). Bottom panel: Ferretome.org database schema related to architecture data modality. The central entity of this schema is the architecture table (linked to a literature table and brain sites table). Architecture can be represented with several parameters and every parameter is supplied with extension and PDC codes.

Similar as for the labeling data modality, architecture data can be extracted from figures as well as from textual descriptions provided in literature reports. Therefore, for this data modality, the same PDC method of specifying the data reliability was employed. Different aspects of PDC_Architecture were gathered from the literature and can be used for an entire brain area as well as for area subcompartments, such as individual cortical layers (**Figure 4**).

For algorithmic services, Ferretome.org uses the implementation of ORT described above. This algorithm was extended in order to process brain architecture in a similar way as transferring labeling information from one brain map to another. This process does not require additional metadata about brain maps relations and transfers all available architectonic parameters simultaneously with the connectivity data. In case of ambiguities, when two different brain map indicate contradictory information about an area or subpart of the area, the algorithm performs a ranking according to extension codes and PDC codes. More reliable data (indicated by better extension codes and PDC codes) is shown first.

### Data Entry Process

In order to comply with established procedures and recommendations for connectome projects (e.g., Bakker et al., 2012), we introduced specific routines for data entry and data modification.

For data entry, a semi-automated pipeline was created with four main steps: (1) systematic literature search and discovery of tract tracing reports, (2) short-listing and queuing of reports for input, (3) input by one DB collator, (4) proofreading by another DB collator.

**FIGURE 5:**
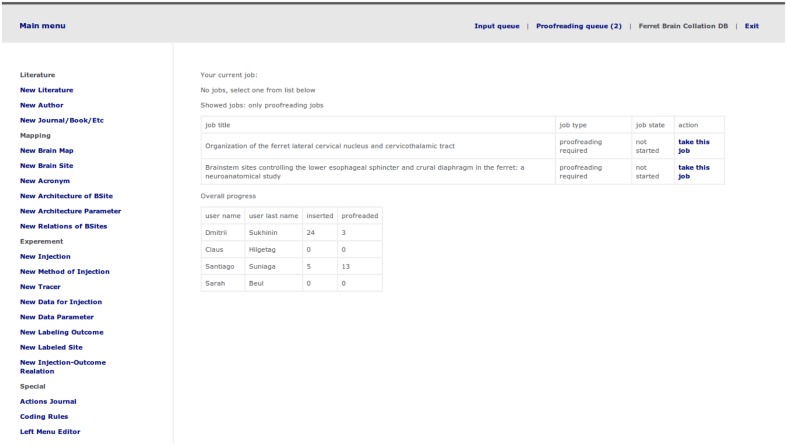
**Task selection and management menu including a short progress report**.

The first and second step are performed outside of the system. During the first step, Ferretome.org curators (trained in brain anatomy) used online search engines such as Google Scholar^6^ and PubMed^7^ to identify ferret brain tract-tracing reports. In the second step, the DB curators, after an initial assessment of a report, decided if it should be added to the database. If so, a curator created a task inside Ferretome.org (**Figure 5**). Moreover, during this step, the DB curators inspected literature references within selected tract-tracing reports and, if these reports used brain maps delineated elsewhere, the respective reports were also selected for entry.

During the third step, the system distributed tasks in such a way that the initial data entry and the proofreading of a tract-tracing report were performed by two different researchers. The step included the detailed evaluation of a tract-tracing report, entering all available data into database and marking the data with extension codes and PDCs. After finishing data entry, the DB curator changed the task status to “finished” in order to proceed to the fourth step. This final step virtually repeats the procedure of the third step, but performed by a different DB curator.

From the perspective of a DB curator, the data entry interface represents a typical web application where user can select necessary section and by means of an input wizard perform entry of data found in tract-tracing report. The data entry pipeline was integrated with a journaling subsystem that keeps track of changes that were made by users for every data modality presented in the system and allows to roll back unwanted changes.

### Use Cases and Technical Information

Although the data entry interface (or ‘back office’) allows navigation across already inserted data, for the convenience of the end users an entire new interface for data browsing was created (‘front office’). This interface interacts with the database in read-only mode. In general, the data browsing interface provides different means of searching information and creating summaries of stored data.

**FIGURE 6:**
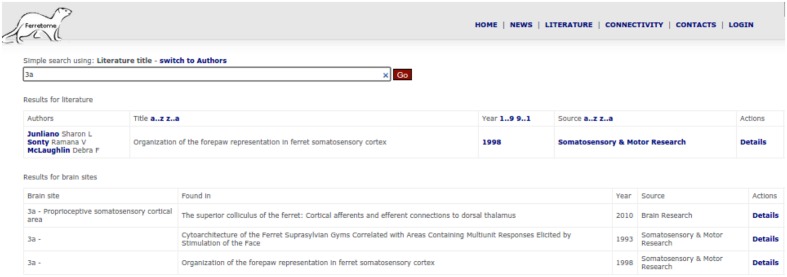
**Search interface of the Ferretome.org front office**.

One way in which this interface can be used is for literature search, where users can try to find data by using bibliographical information (i.e., by the title of a literature report or author names) or by entering the acronym of a brain area (**Figure 6**). Another way to access connectivity data is via the connectivity section (or directly from the literature section), display the entire information provided in a literature report. Ferretome.org automatically maps all available connectivity data from all brain maps present in the DB into a selected map on-the-fly using the ORT algorithm.

At this point the connectivity data can be extracted in two formats, XML and JSON^8^ (more formats are planned, see Discussion) and be further analyzed by approaches such as the brain connectivity toolbox^9^ or neuroVIISAS, mentioned above. A snapshot of the data is provided as a Supplementary File.

Going deeper into technical details, Ferretome.org represents a typical web application with a front-office and a back-office supported by a database. As a database management system, the reliable and free MySQL^10^ was employed and phpMyAdmin^11^ was used to handle the initial creation and editing of tables. The source code and schema of the database are available on GitHub^12^

### System Validation

The Ferretome.org system has so far been used by three members of our lab for data entry. These researchers also provided substantial feedback on the general design of the system. Moreover, this project is being developed as part of a research center collaboration^13^ In this context, we initially presented the conceptual design of the database and as well later preliminary results to other researchers at the center who work experimentally on the ferret brain and who are the main local recipients of this project. These researchers provided helpful feedback on the approach and methodology as well as an approval of the general design of the system.

## Results

**FIGURE 7:**
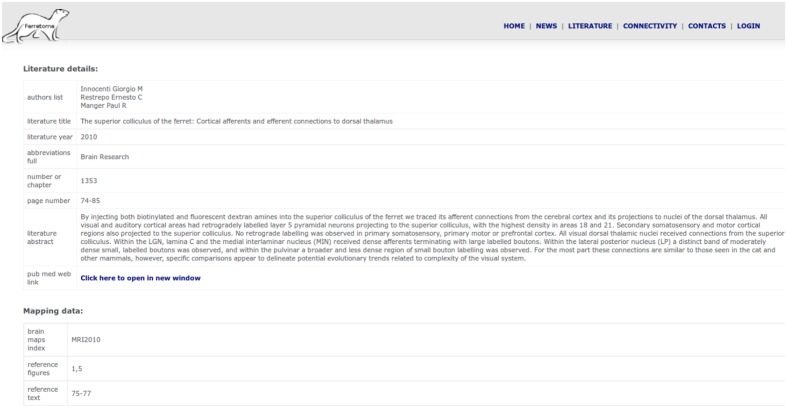
**Summary of inserted data from one literature report**.

Currently, the state of Ferretome.org can be characterized as a beta version. While it integrates all connectivity information for the ferret presently available in the literature (as identified by the DB curators), the available information itself is sparse, so that the information contained in the Ferretome about the brain architecture and macroconnectome of the ferret brain is still limited. Moreover, the relatively small number of anatomical connectivity reports published so far on the ferret covers mostly subcortical connections. However, the database is continuously being populated with newly appearing reports, and we are also working on evaluating still unpublished results of tract-tracing experiments in the ferret as well as performing new experiments. Stored records can be accessed via the web interface (**Figure 7**), where the full summary of inserted data for a given publication is represented as a table. This table can be dynamically extended to display links with other publications (e.g., if brain maps were defined in a different paper and the current record is using these parcellation schemes to describe tract-tracing results).

Using the same interface, the architecture of the brain areas can be obtained directly from the extracted data of a paper, as well as from other records by using the ORT algorithm that transforms connectivity data from one map to another, if relations among parcellations schemes are specified.

At the current point, more than 150 ferret brain papers have been reviewed, 50 them were entered into the database and for 30 of them that contain mapping or connectivity data, the proofreading is finished. These 30 reports contain 20 unique injections sites with 200 labeling sites in both ipsi- and contra-lateral hemispheres of the ferret brain. Architecture data is currently provided for 12 distinct brain areas, primarily for visual and auditory cortex.

## Discussion and Outlook

Differences in the techniques of different neuroanatomical labs and the absence of well-established standards for producing tract-tracing reports create challenges in extracting architecture and connectivity data for systematic computational analysis and cross-species comparative studies. After a review of existing technologies, approaches and methods, it appeared that the most suitable strategy for databasing structural information of the ferret would be a CoCoMac-like approach and database schema. Our motivation was similar to that of the initial CoCoMac development (Stephan, 2013). First, most tract-tracing reports do not provide the exact spatial location of injections sites, but rather employ semantic localisers (such as an injection being made into ‘primary visual cortex’ or ‘area 17’). Second, brain areas in one brain map may be represented very differently in another brain map. In order to build a comprehensive description of ferret brain connectivity, one needs mechanisms to relate one brain area and its connectivity in one parcellation to another brain area in a different parcellation. This transformation is tedious and error-prone if performed by hand, and therefore requires automation. Here, we focused on the problem of how to adapt the CoCoMac approach to the case of the ferret. Our system includes the main features of the CoCoMac approach, including PDC and extension codes as well as the ORT algorithm, but, in addition, we have extended the database schema in order to flexibly accommodate the representation of architecture information of brain areas.

To provide a wide base for the subsequent use of the database, several additional structural parameters were included. One motivation for this approach was the finding that brain connectivity appears to be closely related to the architectonic similarity of cortical areas (e.g., Hilgetag and Grant, 2010; Beul et al., 2014; van den Heuvel et al., 2015). Many literature reports also provide descriptions of brain cytoarchitecture. Such descriptions include the classification of cells, number of layers and sublayers and their density, amongst other features. Such cytoarchitectonic descriptions can be affected by similar problems as connectivity data, because they are usually defined by researchers within their own brain maps and hence need to undergo transformations from one brain map to another.

An important extension of the CoCoMac methodology is to link connectivity data to tools for visualization, analysis and simulation. This perspective is vital not only for understanding functional implications of connectivity, but also for validating data inserted into the database, by providing analytical summaries that can be compared to global models of connectivity organization. Therefore, Ferretome.org should have the functional capacity to extract data of all modalities (including computed brain maps relations) in a variety of formats in order to integrate well with analysis and simulation platforms and (online) atlases, such as the Scalable Brain Atlas^14^ (Bakker et al., 2010). The export of connectivity data in XML and JSON formats is already available and more formats are planned. Integration with atlases will be useful not only for visualization, but can provide new knowledge in the area of comparative studies. For example, co-registering connectivity data with the SVG based Common Atlas format developed by Majka et al. (2012) has facilitated studies in a variety of species, such as opossum and marmoset. Moreover, following the example of the NeuroVIISAS platform (Schmitt and Eipert, 2012), integration with connectivity analysis tools, such as the Brain Connectivity Toolbox^15^, or tools for modeling brain dynamics, like The Virtual Brain^16^ (Ritter et al., 2013), will be provided. This integration will allow characterizing features of structural nodes and circuits and linking them to aspects of brain dynamics and function.

In addition to storing fundamental connectivity and architectural data for the ferret brain, several additions are planned for Ferretome.org that will make access to the data easier or more functional. For example, in the past, an attempt was made to provide CoCoMac with visualization and search automation tools by using external software (Kötter, 2004). To follow the CoCoMac example, in the short term, we are planning the integration of visualization tools that can be deployed at the users’ computer clients (directly in a browser). For example, the use of WebGL technology will allow future integration with a prospective atlas of the ferret brain. Taking into account that Ferretome.org extensively represents the architecture of brain areas, visualization tools could give to users the opportunity to display simultaneously connectivity data and architecture data. Moreover, by analogy with connectivity data, researchers should have the ability to perform a quick survey of architectural data right in their browser. For example, it will be helpful if architectural information on the cellular density and thickness of cortical layers can be read out in standard formats for further offline analysis.

Although in the current state the database does not contain sufficient data to provide connectivity and architectural data for the entire ferret brain, it may already be sufficient for identifying underrepresented brain areas where, for various reasons, tract-tracing studies have not yet been conducted. As soon as new tract-tracing reports appear in literature, the data will be added to Ferretome.org. The collated data do not have to be restricted to cortical connectivity and area-to-area connection systems, but could also include the connectivity of neuromodulatory systems. These systems typically include localized cell populations (such as the orexinergic neurons in the hypothalamus, or the cholinergic and noradrenergic neurons in the pons) that project widely throughout the brain and spinal cord (Dell et al., 2013). These projections are easily identified with immunohistochemistry, and could be readily plotted and quantified with stereological techniques (in terms of regional densities, distribution by cortical layers and neuronal types, etc.) and added to the database. In addition, the quantitative distribution could be determined for the GABAergic neurons stained with parvalbumin, calbindin, and calretinin. Such an effort would provide insight into the organization of inhibitory systems in the brain, in addition to excitatory long-range projections. Although the integration of this type of data is a complex task that requires substantial adaptation of the database structure, it appears feasible and was already partly realized in the neuroVIISAS project (for details see Schmitt and Eipert, 2012).

As a further extension of the concept of this connectivity database, we also consider the possibility of adding the modality of large-scale functional connectivity of the ferret brain, both at rest and during tasks. This idea can be implemented with the same methodology as CoCoMac and Ferretome.org, by providing information on the reliability of data and by transformation of data across different brain maps. A worked example of storing functional connectivity data in the CoCoMac framework was provided by CoCoMacStry, a collation of strychnine-induced functional connectivity of the macaque brain (Stephan et al., 2000a). Ultimately, the structural and functional perspective of connectivity data can be linked through computational modeling platforms.

On the practical side, an efficient implementation and management system is required in order to maintain an up-to-date database that is quick and functional as well as easy to handle by administrators and users. One way of achieving this aspect is by providing constant web access to all parts of the database. In this case, data in the database can be reviewed not only by the database collators, but also external experts. In the long-term, an important goal is the involvement of the scientific community, in particular of experimental neuroanatomists, for contributing new data or validating the information already existing in the database. This step is essential for verifying the overall consistency of the data and facilitating the dialog among all parties interested in ferret brain structure and function. Thus, the system has to be designed in such a way that it is accessible and appealing to experimentalists studying the ferret brain. Based on this idea of community participation, one of the options for increasing the value of the databasing project is to have the ability to store the raw data (such as images, or detailed quantitative information) taken directly from experiments. From the technological point of view, this is a challenging task that requires development of special storage subsystems and algorithms for data access as well as data protection methods at different levels of data access, public and private.

In summary, here we introduced Ferretome.org, a ferret brain macro-connectivity and architecture database. This project is built upon the experience of a previous generation of neuroinformatics project such as XNAT, BAMS, NeuroVIISAS, and in particular CoCoMac. Specifically, Ferretome.org inherited from CoCoMac the basic methodology and philosophy of objectivity and reproducibility, and follows the same data collation rules and standards. In addition, we extended the basic CoCoMaC methodology in order to capture architectural data that provide an important context for connectivity data. Currently, we are moving toward extensive population of the database with newly published results and thus hope to make a useful contribution to the study of ferret brain structure and function.

## Author Contributions

All authors listed have made substantial, direct and intellectual contribution to the work, and approved it for publication.

## Conflict of Interest Statement

The authors declare that the research was conducted in the absence of any commercial or financial relationships that could be construed as a potential conflict of interest.

## Footnotes

1 uberon.github.io

2 https://bams1.org.

3 https://www.php.net

4 https://www.mysql.com.

5 http://cocomac.g-node.org

6 http://scholar.google.com

7 http://www.ncbi.nlm.nih.gov/pubmed

8 json.org

9 http://www.brain-connectivity-toolbox.net/

10 www.mysql.com

11 www.phpmyadmin.net

12 github.com/ICNS-uke/Ferretome.git.

13 www.sfb936.net.

14 scalablebrainatlas.org

15 www.brain-connectivity-toolbox.net

16 www.thevirtualbrain.org
